# Retrospective assessment of cyclin‐dependent kinase 5 mRNA and protein expression and its association with patient survival in breast cancer

**DOI:** 10.1111/jcmm.15268

**Published:** 2020-04-30

**Authors:** Behnaz Saidy, Emad A. Rakha, Andrew R. Green, Ian O. Ellis, Stewart G. Martin, Sarah J. Storr

**Affiliations:** ^1^ Division of Cancer and Stem Cells Nottingham Breast Cancer Research Centre School of Medicine University of Nottingham Biodiscovery Institute Nottingham UK

**Keywords:** breast cancer, Cdk5, prognosis

## Abstract

Cyclin‐dependent kinase 5 (Cdk5) is an atypical member of the cyclin‐dependent kinase family and functions as a serine/threonine kinase that can be activated by non‐cyclin binding activators p35 or p39. Cdk5 expression and activity has been linked with the development and progression of cancer; however, its expression in breast cancer has not been fully described. Protein expression of Cdk5 was determined in a large cohort of early‐stage invasive breast cancer tumours (n = 1110) with long‐term follow‐up data using immunohistochemistry. Expression of *CDK5* mRNA was assessed in the METABRIC cohort (n = 1980). Low nuclear and cytoplasmic expression of Cdk5 expression was significantly associated with shorter breast cancer‐specific survival (*P* = .004 and *P* = .001, respectively). Importantly, low nuclear and cytoplasmic expression of Cdk5 remained associated with survival in multivariate analysis, including potentially confounding factors (hazard ratio (HR) = 0.612, 95% confidence interval (CI) = 0.418‐0.896, *P* = .011 and HR = 0.507, 95% CI = 0.318‐0.809, *P* = .004, respectively). In addition, low nuclear and cytoplasmic expression of Cdk5 was significantly associated with clinicopathological criteria associated with adverse patient prognosis. Low *CDK5* mRNA expression was associated with shorter patient survival (*P* = .005) in the METABRIC cohort; no associations between copy gain or loss and survival were observed. These data suggest that low Cdk5 expression is associated with poor clinical outcome of breast cancer patients and may be of clinical relevance.

## 1 INTRODUCTION

Cyclin‐dependent kinase 5 (Cdk5) is a proline‐directed serine/threonine kinase and an atypical member of the cyclin‐dependent kinase family. Unlike most of the conventional cyclin‐dependent kinases, Cdk5 is predominantly expressed in neurons and can be activated by binding of non‐cyclin activators p35 or p39. Cdk5 plays a crucial role in regulating the cytoarchitecture of the central nervous system; however, it is clear that Cdk5 can play a role in cell types other than neurons and in the pathogenesis of certain diseases, such as cancer and Alzheimer's disease. Cdk5 functions in the development and progression of cancers through modulation of key pathways such as DNA repair and cellular migration.

Cdk5 was first described in 1992,^1^ and its function and numerous substrates have subsequently been described (reviewed in Ref. [2]). Monomeric Cdk5 is inactive and requires binding of p35 or p39 to become activated, and it also functions in a negative feedback pathway through phosphorylation of p35 leading to its ubiquitination and subsequent degradation.^3^

In neurons, Cdk5 functions in numerous important cellular pathways; for example, phosphorylation of apurinic/apyrimidinic endonuclease 1 (Ape1) by Cdk5/p35 complexes reduces the AP endonuclease activity of Ape1, an enzyme critical in the base excision repair pathway, to allow DNA damage to accumulate.^4^ Cdk5 also phosphorylates focal adhesion kinase (FAK) allowing regulation of a microtubule fork that results in neuronal migration.^5^ In cancer, Cdk5 participates in numerous pathways associated with tumour progression. Cdk5 can influence cellular migration in cancer, such as phosphorylation of FAK, promoting the formation of F‐actin bundles to facilitate epithelial to mesenchymal transition and motility.^6^ In addition, phosphorylation of GIV by Cdk5 is to play a role in the migration‐proliferation dichotomy,^7^ and in breast cancer, Cdk5 phosphorylation of adducin‐1 is important for epidermal growth factor‐induced cell migration and invasion.^8^

In cancer, high Cdk5 protein and mRNA expression is associated with clinicopathological features associated with a poor prognosis or adverse survival in a number of tumour types. In lung cancer, high *CDK5* mRNA expression has been associated with survival, and protein expression is associated with numerous clinicopathological criteria associated with a poor prognosis.^9^, ^10^

Studies have assessed the association between Cdk5 and survival; however, this has been in tumour types other than breast. In colorectal cancer, high Cdk5 expression is associated with poor prognosis,^11^ and in gastric cancer, low Cdk5 expression has been associated with shorter patient survival.^12^ In breast cancer, Cdk5 expression has been associated with prognostic factors associated with worse patient prognosis; however, no association with survival was reported.^6^ We sought to determine the frequency of Cdk5 overexpression in a large cohort of early‐stage invasive breast cancers to determine whether Cdk5 expression was associated with poor patient survival and test associations with clinicopathological criteria.

## 2 METHODS

### 2.1 Patient cohorts

A total of 1110 early‐stage invasive, primary breast cancer patients were included in this study and were treated between 1998 and 2006 at Nottingham University Hospitals. All patients underwent a wide local excision or mastectomy, which was determined by disease characteristics or patient choice; this was then followed by radiotherapy, if indicated. Oestrogen receptor (ER) positivity/negativity, Nottingham Prognostic Index (NPI) value and menopausal status were used to determine whether a patient received systemic adjuvant treatment. Patients with an NPI index 3.4 or greater were candidates for CMF combination chemotherapy (cyclophosphamide, methotrexate and 5‐fluorouracil) if they were ER negative or premenopausal; and hormone therapy if they were ER positive. Breast cancer‐specific survival was calculated as the time between death resultant from breast cancer and primary surgery, and the reverse Kaplan‐Meier method was used to calculate the median patient follow‐up as 148 months. REMARK criteria were used to report this study.^13^

The molecular taxonomy of breast cancer international consortium (METABRIC) data series was also used (n = 1980) with information about the data set published elsewhere.^14^ Tumours were collected between 1977 and 2005 from five centres in the UK and Canada. The reverse Kaplan‐Meier method was used to determine the median patient follow‐up as 141 months. ER‐negative and/or lymph node‐positive patients did not receive adjuvant chemotherapy, whereas almost all ER‐negative and lymph node‐positive patients did. Trastuzumab was not given to any patients with HER2 overexpression. In brief, RNA and DNA were isolated from samples and hybridized to the Affymetrix SNP 6.0 and Illumina HT‐12 v3 platforms for genomic and transcriptional profiling.

### 2.2 Immunohistochemistry

Tissue microarrays were used for immunohistochemistry and were composed of 0.6 mm tissue cores taken from representative tumour areas which were determined using Haematoxylin and eosin‐stained tissue. Leica Novolink Polymer Detection kit was used according to the manufacturers’ instructions and has been described previously^15^; briefly, the slides were heated on a 60°C hotplate for 10 minutes prior to deparaffinization and rehydration in xylene, ethanol and then water. Tissue was microwaved in 0.01 mol L^−1^ sodium citrate buffer for 10 minutes at 750 W followed by 10 minutes at 450 W. Novolink Peroxidase Block was incubated on the tissue for five minutes prior to washing with Tris‐buffered saline (TBS) and followed by incubation with Novolink Protein Block solution. Cdk5 mouse monoclonal antibody (Cell Signalling 12134) was incubated on the tissue microarray at room temperature for one hour, once diluted 1:500. TBS was used to wash the tissue prior to incubation with Novolink Post Primary solution for 30 minutes followed by washing and incubation with Novolink Polymer solution for 30 minutes. The chromogenic substrate for developing immunohistochemical reactions was 3, 3′diaminobenzidine, and tissue was then counterstained with Haematoxylin. Following staining, tissue was dehydrated in sequential washes of ethanol and xylene before mounting using DPX. A breast tumour composite section, comprised of grade one and two early‐stage invasive tumours, was used as a positive and negative control and was included in each staining run; negative controls had primary antibody omitted.

Slides were assessed following scanning using a Nanozoomer Digital Pathology Scanner (Hamamatsu Photonics) at 200× magnification. Cytoplasmic staining was manually assessed using an immunohistochemical H‐score, where the percentage area of each staining intensity (none (0), weak (1), medium (2) or strong (3)) was assessed. Nuclear staining was manually assessed in a semi‐quantitative manner, where the percentage of tumour cells that demonstrated any intensity of staining was determined. The final H‐score was assessed multiplying the intensity by the percentage of positive cells.

### 2.3 Statistical analyses

IBM SPSS (version 24) was used to conduct statistical analysis. The final H‐score and *CDK5* mRNA expression was stratified into low and high expression using X‐tile software based on breast cancer‐specific survival.^16^ Double assessment was performed in over 30% of cases, with, with both assessors blinded to both each other's H‐score and the clinical outcome of the patients. Intraclass correlation coefficients (single measure) were 0.958 and 0.912 for Cdk5 cytoplasmic and nuclear scores, respectively, both indicating good concordance between scorers. The Pearson chi‐squared test of association enabled assessment of the connection between clinicopathological variables and categorized protein expression. The Kaplan‐Meier method was used to plot survival curves, with significance determined using the log‐rank test. All differences were considered statistically significant at the level of *P* ≤ .05. DARPP‐32 staining and scoring has been reported previously.^17^

## 3 RESULTS

### 3.1 Cdk5 expression in invasive breast cancer

**FIGURE 1:**
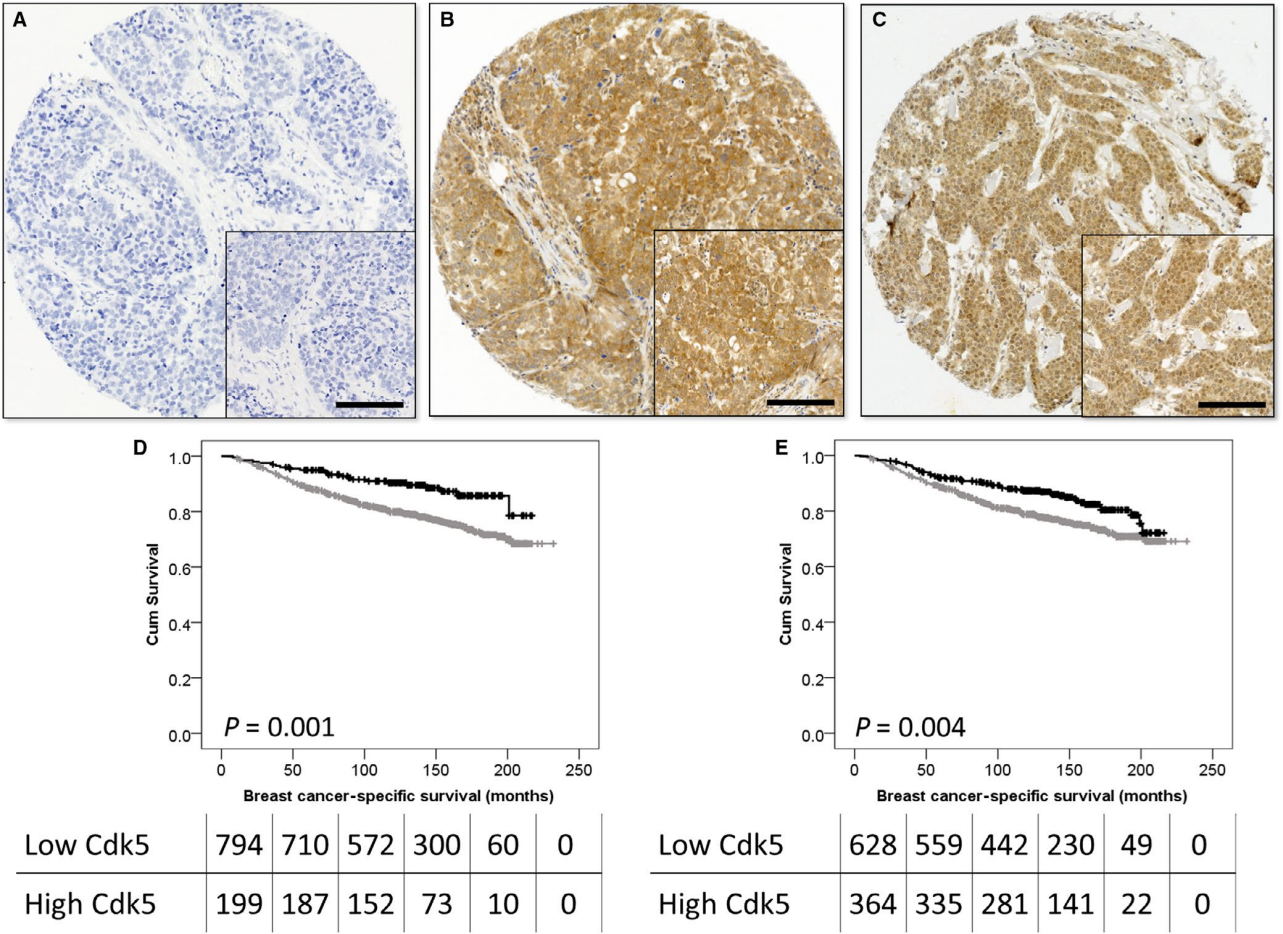
Representative photomicrographs of Cdk5 immunohistochemistry demonstrating absence of Cdk5 staining (A), cytoplasmic staining of Cdk5 (B), and cytoplasmic/nuclear staining of Cdk5 (C) shown at 100× magnification with a 200× magnification inset box; scale bar represents 50 µm. Kaplan‐Meier analysis of Cdk5 expression, where the impact of low (grey line) and high (black line) expression within the cytoplasm (D) and nucleus (E) are shown. Numbers shown below the Kaplan‐Meier survival curves are the number of patients at risk at the specified month

Cytoplasmic and nuclear Cdk5 staining varied from strong to weak, and representative photomicrographs are shown in Figure 1A‐C. Nuclear Cdk5 expression was significantly correlated with cytoplasmic Cdk5 expression (*P* < .001, *r* ^2^ = .569). Nuclear Cdk5 expression had a median H‐score of 5 and ranged from 0 to 85; an X‐tile calculated cut point of H‐score 9 was used, and 63.9% of cases (710/1110) demonstrated low expression. Cytoplasmic Cdk5 expression had a median H‐score of 105 and ranged from 0 to 240; an X‐tile calculated cut point of H‐score 159 was used, and 50.6% of cases (562/1110) demonstrated low expression.

### 3.2 Cdk5 expression and clinicopathological parameters

Associations between cytoplasmic and clinicopathological variables and nuclear Cdk5 expression were assessed (Table 1). Low nuclear Cdk5 expression was significantly associated with larger tumour size (*χ* ^2^ = 7.858, *df* = 1, *P* = .005), higher tumour grade (*χ* ^2^ = 7.630, *df* = 2, *P* = .022), mitosis (*χ* ^2^ = 7.702, *df* = 2, *P* = .021), worse NPI value (*χ* ^2^ = 8.386, *df* = 2, *P* = .015), ER‐negative tumours (*χ* ^2^ = 6.754, *df* = 1, *P* = .009) and triple receptor‐negative tumours (*χ* ^2^ = 5.927, *df* = 1, *P* = .015; Table 1). Low cytoplasmic expression of Cdk5 was significantly associated with a higher NPI values (*χ* ^2^ = 7.334, *df* = 2, *P* = .026; Table 1).

**TABLE 1:** The associations between clinicopathological variables and nuclear and cytoplasmic expression of Cdk5 in a large cohort of breast cancer patients

	Cytoplasmic Cdk5 expression			Nuclear Cdk5 expression		
	Low	High	*P* value	Low	High	*P* value
Age						
<50 years	289 (26.3%)	70 (6.4%)	.748	234 (21.4%)	124 (11.3%)	.500
≥50 years	588 (53.6%)	150 (13.7%)		467 (42.6%)	271 (24.7%)	
Size						
<2.0cm	507 (46.3%)	143 (13.0%)	.055	393 (35.9%)	256 (23.4%)	**.005**
≥2.0cm	369 (33.7%)	77 (7.0%)		307 (28.0%)	139 (12.7%)	
Grade						
1	131 (12.0%)	41 (3.7%)	.127	102 (9.3%)	70 (6.4%)	**.022**
2	331 (30.2%)	91 (8.3%)		256 (23.4%)	166 (15.2%)	
3	414 (37.8%)	88 (8.0%)		342 (31.2%)	159 (14.5%)	
Pleomorphism						
1	12 (1.1%)	3 (0.3%)	.909	8 (0.7%)	7 (0.6%)	.316
2	247 (22.8%)	65 (6.0%)		191 (17.6%)	121 (11.2%)	
3	609 (56.1%)	149 (13.7%)		494 (45.6%)	263 (24.3%)	
Mitosis						
1	398 (36.7%)	113 (10.4%)	.224	306 (28.3%)	205 (18.9%)	**.021**
2	170 (15.7%)	41 (3.8%)		137 (12.7%)	74 (6.8%)	
3	299 (27.6%)	63 (5.8%)		249 (23.0%)	112(10.3%)	
Vascular invasion						
Definite	265 (24.2%)	53 (4.8%)	.072	210 (19.2%)	107 (9.8%)	.308
No/probable	611 (55.7%)	167 (15.2%)		490 (44.7%)	288 (26.3%)	
Stage						
1	549 (50.1%)	142 (13.0%)	.167	440 (40.2%)	251 (22.9%)	.780
2	234 (21.4%)	64 (5.8%)		188 (17.2%)	109 (10.0%)	
3	92 (8.4%)	14 (1.3%)		71 (6.5%)	35 (3.2%)	
NPI						
Good (≤3.4)	274 (25.0%)	85 (7.8%)	**.026**	208 (19.0%)	151 (13.8%)	**.015**
Intermediate (3.41‐5.4)	444 (40.6%)	110 (10.1%)		370 (33.9%)	183 (16.7%)	
Poor (>5.4)	156 (14.3%)	25 (2.3%)		121 (11.1%)	60 (5.5%)	
ER status						
Negative	186 (16.9%)	38 (3.5%)	.186	160 (14.6%)	64 (5.8%)	**.009**
Positive	691 (62.9%)	183 (16.7%)		542 (49.4%)	331 (30.2%)	
PgR status						
Negative	373 (36.2%)	83 (8.1%)	.103	303 (29.5%)	153 (14.9%)	.243
Positive	445 (43.2%)	128 (12.4%)		360 (35.0%)	212 (20.6%)	
HER2 status						
Negative	748 (72.3%)	192 (18.6%)	.769	603 (58.4%)	336 (32.5%)	.628
Positive	76 (7.4%)	18 (1.7%)		58 (5.6%)	36 (3.5%)	
Triple‐negative status						
Negative	728 (67.6%)	189 (17.5%)	.365	572 (53.2%)	344 (32.0%)	**.015**
Positive	132 (12.3%)	28 (2.6%)		116 (10.8%)	44 (4.1%)	

Note

The *P* values shown in the table are resultant from Pearson's chi‐squared test of association; *P* < .05 are highlighted in bold. NPI is Nottingham Prognostic Index, ER is oestrogen receptor, and PgR is progesterone receptor.

### 3.3 Cdk5 expression and breast cancer‐specific survival

Both low nuclear and cytoplasmic expressions of Cdk5 were associated with adverse breast cancer‐specific survival (*P* = .004 and *P* = .001, respectively; Figure 1D,E). Low nuclear Cdk5 expression was significantly associated with survival (hazard ratio (HR) = 0.612, 95% confidence interval (CI) = 0.418‐0.896, *P* = .011) when confounding prognostic factors were included in multivariate analysis (including tumour size, tumour grade, stage, NPI, ER status, PR status, HER2 status and vascular invasion [all with individual log‐rank statistics of *P < *.001]). Low cytoplasmic Cdk5 expression also remained significantly associated with poor survival (HR = 0.507, 95% CI = 0.318‐0.809, *P* = .004) when the same potentially confounding prognostic factors were included in the multivariate analysis.

### 3.4 Cdk5 expression and ER status

**FIGURE 2:**
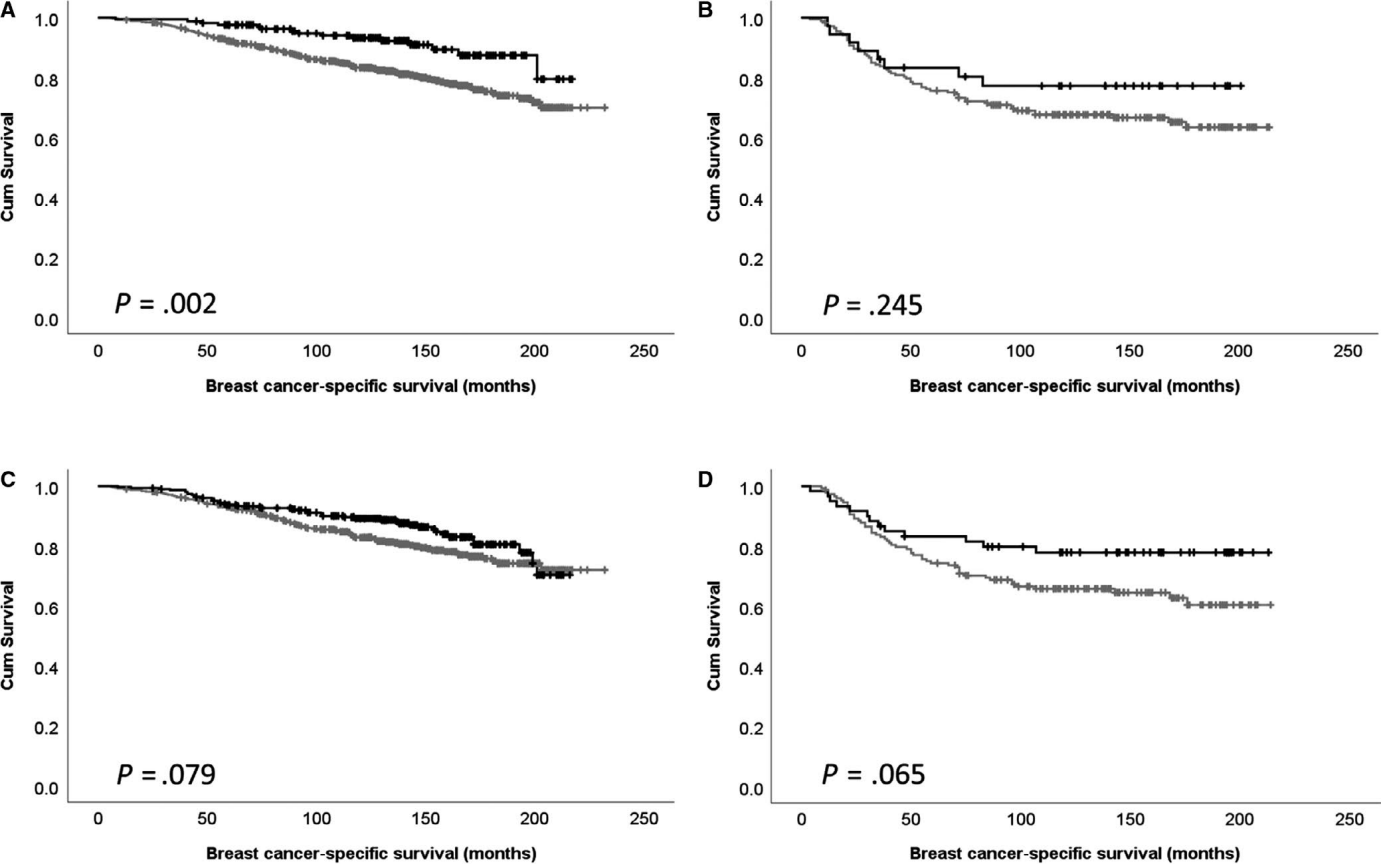
Kaplan‐Meier analysis of cytoplasmic Cdk5 protein expression in oestrogen receptor (ER)‐positive disease (A) and ER‐negative disease (B); and nuclear Cdk5 expression in ER‐positive disease (C) and ER‐negative disease (D), where low expression (grey line) and high expression (black line) are shown

Cdk5 expression is association with survival was further assessed in ER‐positive and negative disease. Cytoplasmic expression of Cdk5 was significantly associated with survival of ER‐positive patients (*P* = .002), but not ER‐negative patients (*P* = .245; Figure 2A,B). There was no association between nuclear Cdk5 expression and patient survival in ER‐positive or negative subgroups (*P* = .079 and *P* = .065, respectively; Figure 2C,D).

### 3.5 Cdk5 expression and expression of DARPP‐32

As Cdk5 is linked with DARPP‐32, we assessed for correlation between Cdk5 and previously determined DARPP‐32 expression.^17^ Cytoplasmic Cdk5 expression was statistically correlated with cytoplasmic DARPP‐32 expression, although demonstrating a weak biological association (*P* = .004, *r* ^2^ = .090). A similar finding was observed between nuclear DARPP‐32 and nuclear Cdk5 expression (*P* = .001, *r* ^2^ = 0.103).

### 3.6 CDK5 mRNA expression and patient survival

**FIGURE 3:**
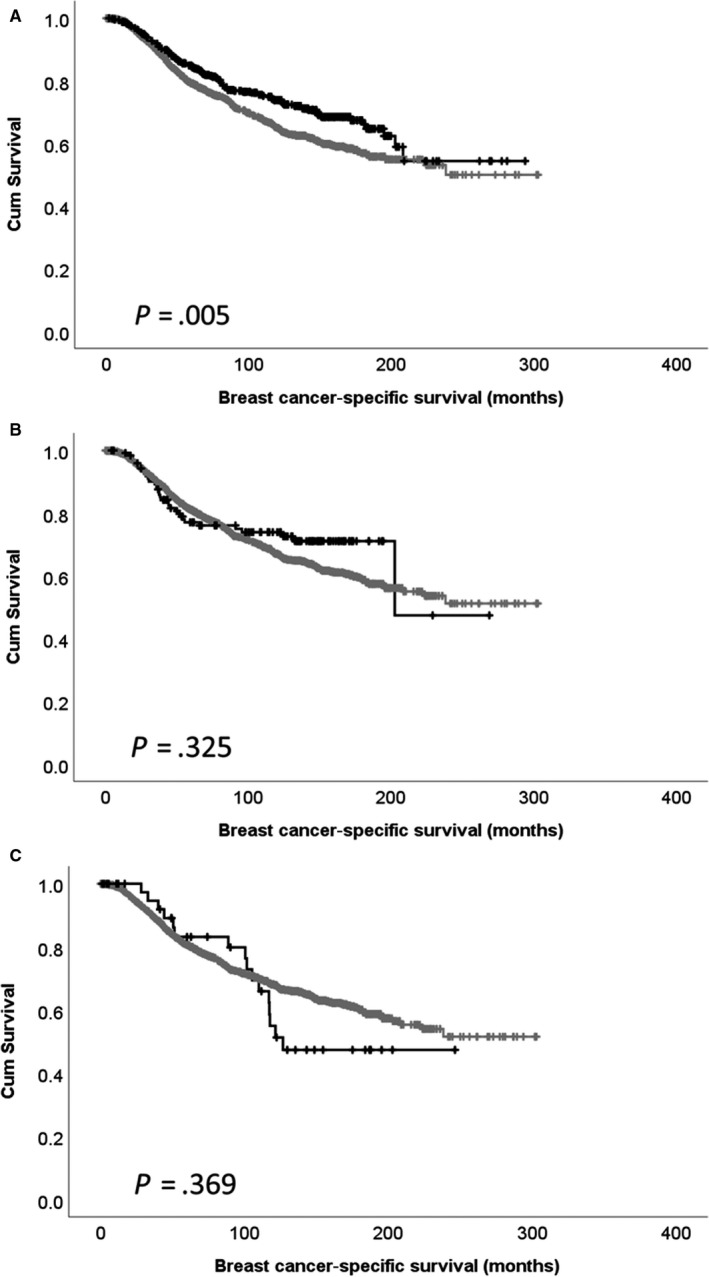
Kaplan‐Meier analysis of CDK5 expression (A) copy gain (B) and copy loss (C). In panel (A), the low expression (grey line) and high expression (black line) are shown. In panel (B), the black line indicates copy gain, and in panel C, the black line indicates copy loss

**FIGURE 4:**
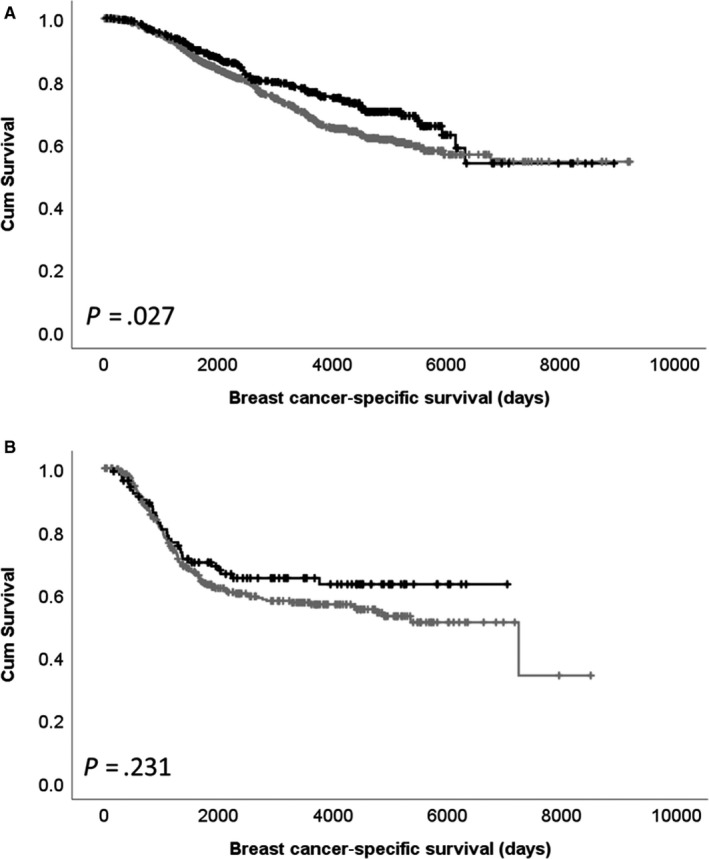
Kaplan‐Meier analysis of *CDK5* mRNA expression in oestrogen receptor (ER)‐positive disease (A) and ER‐negative disease (B), where low expression (grey line) and high expression (black line) are shown

Expression of *CDK5* mRNA was assessed for associations with available clinicopathological data (Table 2); high *CDK5* mRNA expression was significantly associated with higher tumour grade (*χ* ^2^ = 12.898, *df* = 2, *P* = .002), luminal B PAM50 subtype (χ^2^ = 88.278, *df* = 5, *P* < .001), ER‐positive disease (*χ* ^2^ = 8.588, *df* = 1, *P* = .003) and HER2‐negative disease (χ^2^ = 9.922, *df* = 1, *P* = .002). Low expression of *CDK5* mRNA was significantly associated with shorter patient survival (*P* = .005; Figure 3A). No association between *CDK5* copy gain or copy loss with patient survival was observed (Figure 3B,C). *CDK5* mRNA expression was associated with adverse survival in ER‐positive patients (*P* = .027), but not ER‐negative patients (*P* = .231; Figure 4A,B).

**TABLE 2:** Associations between available clinicopathological variables and *CDK5* mRNA expression in the METABRIC cohort

	*CDK5* mRNA expression		
	Low	High	*P* value
Grade			
1	141 (7.5%)	29 (1.5%)	**.002**
2	548 (29.0%)	222 (11.7%)	
3	661 (34.9%)	291 (15.4%)	
Stage			
1	374 (25.7%)	127 (8.7%)	.544
2	589 (40.5%)	236 (16.2%)	
3	89 (6.1%)	29 (2.0%)	
4	7 (0.5%)	3 (0.2%)	
ER status			
Negative	365 (18.4%)	109 (5.5%)	**.003**
Positive	1055 (53.3%)	451 (22.8%)	
HER2 status			
Negative	1222 (61.7%)	511 (25.8%)	**.002**
Positive	198 (10.0%)	49 (2.5%)	
PgR status			
Negative	671 (33.9%)	269 (13.6%)	.754
Positive	749 (37.8%)	291 (14.7%)	
PAM50 subtype			
Basal	240 (12.1%)	89 (4.5%)	<.001
HER2	185 (9.3%)	55 (2.8%)	
LumA	535 (27.0%)	183 (9.2%)	
LumB	278 (14.0%)	210 (10.6%)	
NC	5 (0.3%)	1 (0.1%)	
Normal	177 (8.9%)	22 (1.1%)	

Note

The *P* values shown in the table are resultant from Pearson's chi‐squared test of association, and *P* < .05 are highlighted in bold. ER is oestrogen receptor, and PgR is progesterone receptor.

## 4 DISCUSSION

In the current study, we demonstrate that, in a large cohort of breast cancer patients, low nuclear and cytoplasmic expression of Cdk5 is significantly associated with adverse disease‐specific survival (*P* = .004 and *P* = .001, respectively). Importantly, low nuclear and cytoplasmic Cdk5 expression was significantly associated with survival in multivariate assessment, when potentially confounding factors were included (*P* = .011 and *P* = .004, respectively). In addition to protein expression, low *CDK5* mRNA expression was associated with shorter patient survival in the METABRIC cohort (*P* = .005).

Studies have assessed the expression of Cdk5 and its association with survival in cancer; however, this has been in tumour types other than breast. In gastric cancer, low Cdk5 expression has been associated with adverse survival in 240 patient specimens.^12^ It is important to note that the current study assessed Cdk5 expression, which is not an indication of Cdk5 activity. If Cdk5 expression is directly linked with protein activity in breast cancer, then this study indicates that a Cdk5 inhibition strategy may not be appropriate.

A number of studies have assessed levels of *CDK5* mRNA and associations with survival in breast cancer. High expression of *CDK5* mRNA has been shown to be associated with adverse metastasis‐free survival in a large study of 456 patients; patient clinicopathological variables and breast cancer‐specific survival were not described, so a direct comparison with the current study cannot be made.^18^ Furthermore, using a bioinformatics data mining process, *CDK5* mRNA expression was not associated with overall survival in a study containing 1402 breast cancer patients.^10^ In the current study, low *CDK5* mRNA expression is associated with shorter breast cancer‐specific survival of a large well‐annotated cohort of breast cancer patients (*P* = .005). In addition to breast cancer, *CDK5* mRNA expression is important in other tumour types; in blood cancers, low *CDK5* mRNA expression has been shown to be associated with adverse survival (n = 53) and high *CDK5* expression has been associated with shorter overall survival in lung cancer.^10^

Cdk5 has been shown to play an important role in numerous pathways linked with cancer, with low and high expression associated with survival and or clinicopathological criteria dependent upon tumour type. In breast cancer, high expression of Cdk5, determined by immunoblotting, is associated with ER‐negative tumours and basal‐like tumours in 108 patients; however, no information on survival was available in this study.^6^ In the current study, we also demonstrated an association with ER status, with low nuclear Cdk5 expression associated with ER‐positive tumours. In addition, low cytoplasmic Cdk5 expression was associated with shorter survival of ER‐positive patients (*P* = .002), but not ER‐negative patients. This association was also observed with *CDK5* mRNA expression and survival of ER‐positive patients in the METABRIC cohort (*P* = .027).

## 5 CONCLUSION

Low nuclear and cytoplasmic expression of Cdk5 is associated with poor breast cancer‐specific survival of breast cancer patients; in addition to survival, Cdk5 expression was associated with a number of clinicopathological criteria indicative of poor prognosis. Importantly, low nuclear and cytoplasmic Cdk5 expression remained significantly associated with survival in multivariate assessment, when potentially confounding factors were included (*P* = .011 and *P* = .004 respectively). In addition to protein expression, low *CDK5* mRNA expression was associated with shorter survival of patients in the METABRIC cohort. These findings warrant further investigation in larger patient cohorts, but indicate that Cdk5 expression may be of clinical relevance in breast cancer.

## CONFLICT OF INTEREST

There is no conflict of interest to declare.

## AUTHOR CONTRIBUTIONS

BS and SS conducted the studies and collected the data; ER, IE and AG provided tissue and clinical information; SS conducted statistical analysis; SGM and SS conceived the study; SS wrote the manuscript; and all authors approved the manuscript for submission.

## ETHICAL APPROVAL

This study was approved by the Nottingham Research Ethics Committee 2 (Reference title: Development of a molecular genetic classification of breast cancer). All procedures performed in studies involving human participants were in accordance with the ethical standards of the institutional and/or national research committee, and with the 1964 Helsinki declaration and its later amendments or comparable ethical standards.

## Data Availability

Immunohistochemistry data sets are available from the corresponding author by request.
